# Antiplasmodial Activity of *p*-Substituted Benzyl Thiazinoquinone Derivatives and Their Potential against Parasitic Infections

**DOI:** 10.3390/molecules25071530

**Published:** 2020-03-27

**Authors:** Marcello Casertano, Marialuisa Menna, Caterina Fattorusso, Nicoletta Basilico, Silvia Parapini, Marco Persico, Concetta Imperatore

**Affiliations:** 1 Department of Pharmacy, University of Naples “Federico II”, Via D. Montesano 49, 80131 Napoli, Italy; marcello.casertano@unina.it (M.C.); mlmenna@unina.it (M.M.); caterina.fattorusso@unina.it (C.F.); marco.persico@unina.it (M.P.); 2 Italian Malaria Network, Centro Interuniversitario di Ricerche Sulla Malaria (CIRM), Department of Pharmacy, University of Naples “Federico II”, Via D. Montesano 49, 80131 Napoli, Italy; 3 Dipartimento di Scienze Biomediche, Chirurgiche e Odontoiatriche, Università di Milano, Via Pascal 36, 20133 Milan, Italy; nicoletta.basilico@unimi.it; 4 Dipartimento di Scienze Biomediche per la Salute, Università di Milano, Via Pascal 36, 20133 Milan, Italy; silvia.parapini@unimi.it

**Keywords:** thiazinoquinones, *Plasmodium falciparum*, quinone-derived antimalarial agents, marine inspired compounds, cytotoxicity, antiparasitic agents

## Abstract

Malaria is a life-threatening disease and, what is more, the resistance to available antimalarial drugs is a recurring problem. The resistance of *Plasmodium falciparum* malaria parasites to previous generations of medicines has undermined malaria control efforts and reversed gains in child survival. This paper describes a continuation of our ongoing efforts to investigate the effects against *Plasmodium falciparum* strains and human microvascular endothelial cells (HMEC-1) of a series of methoxy *p*-benzyl-substituted thiazinoquinones designed starting from a pointed antimalarial lead candidate. The data obtained from the newly tested compounds expanded the structure–activity relationships (SARs) of the thiazinoquinone scaffold, indicating that antiplasmodial activity is not affected by the inductive effect but rather by the resonance effect of the introduced group at the *para* position of the benzyl substituent. Indeed, the current survey was based on the evaluation of antiparasitic usefulness as well as the selectivity on mammalian cells of the tested *p*-benzyl-substituted thiazinoquinones, upgrading the knowledge about the active thiazinoquinone scaffold.

## 1. Introduction

The World Health Organization (WHO) considers human malaria as one of the major public health burdens, affecting primarily populations in tropical and subtropical countries [1,2]. Pregnant women and children are particularly vulnerable, but malaria infection is also a severe problem for travelers who come into endemic regions and for immunocompromised people. The decrease in the number of infected people, around 20 million fewer than 2010, is clear evidence of the great efforts made to fight this parasite. Nevertheless, in the timeframe 2015–2017, no significant progress has been recorded in term of a reduction of global malaria cases, with a remarkable percentage, approximatively 40%, of the world’s population still at risk of infection [2]. An additional setback is represented by multiparasitism; often, the malaria patient is co-infested by other parasite species, such as *Schistosoma* and *Leishmania*, classified as neglected tropical diseases (NTDs) due to the absence of efficient and consistent politics based on prevention and a consequent lack of investments by pharmaceutical industries [3].

**Figure 1:**
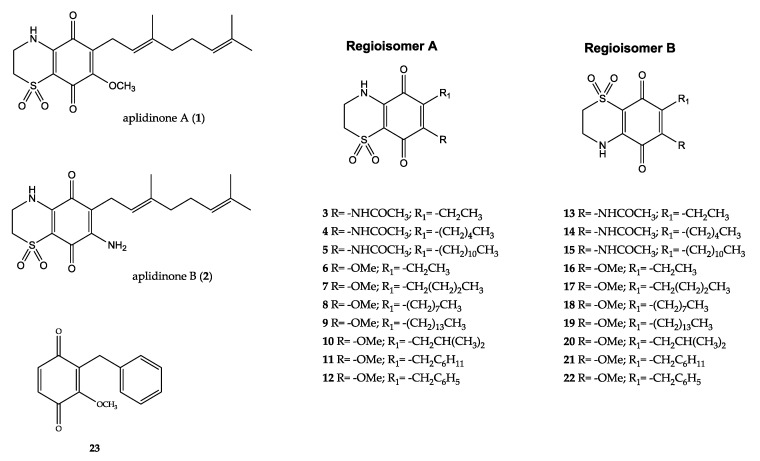
Structures of the marine secondary metabolites aplidinones A (**1**) and B (**2**), and of the synthetic methoxy derivatives **3**–**23**.

In the frame of our studies aiming to explore new chemical entities that can be employed for the discovery of new drugs against malaria and other parasitic diseases [4,5,6,7,8,9], we have recently identified the bicyclic 1,1-dioxo-1,4-thiazine system fused to the quinone ring as a new scaffold active against *P. falciparum* and *Schistosoma mansoni* [4,5,6,7,8,9] exploiting natural marine metabolites as model compounds [10,11,12,13,14]. This work, based on the model of natural marine metabolites, aplidinone A and B (**1** and **2**), has yielded a chemical library of synthetic thiazinoquinones (**3**–**22**, Figure 1), which was funneled into a large screening for the search of antimalarial agents [4,6,9]. Many of these compounds have been demonstrated as effective antimalarials, but the research program has been focused on the methoxy-substituted compounds (**6**–**12** and **16**–**22**, Figure 1). Structure–activity relationship (SAR) studies evidenced that in this group of thiazinoquinones, only the compounds in which the methoxy substituent on the quinone ring is on the same side of the nitrogen of the thiazine ring (regioisomers B) were active, indicating this regiochemistry of the bicyclic moiety is a crucial requirement for the antiplasmodial activity [4,6]. Thus, the synthetic protocol designed for the production of methoxy thiazinoquinones with different alkyl side chains has been improved to make it as selective as possible in favor of the active regioisomer [4,6]. Among the tested thiazinoquinones, we have identified a lead candidate, compound **22**, featuring a benzyl alkyl chain; it was found to be active in the range of low-micromolar concentrations against both chloroquine-sensitive (D10) and -resistant (W2) *P. falciparum* strains, and exhibited a good selectivity index (SI) on the tested mammalian cells (>25). The key role of the thiazinoquinone heterocyclic moiety for the antiplasmodial activity was confirmed by the absence of activity showed by compound **23**, the corresponding quinone of **22** lacking the thiazine ring (Figure 1) [6]. The proposed antimalarial mechanism of action for **22** has been related to its capability to form a stable radical on the benzyl substituent that impairs the *Plasmodium* defense in the heme group detoxification pathway [6]. Definitively, overall, these results supported the eligibility of the thiazinoquinone as a new redox active scaffold, as an alternative to naphthoquinones [15], to be explored for its properties as a lead for the development of novel antimalarials.

**Figure 2:**
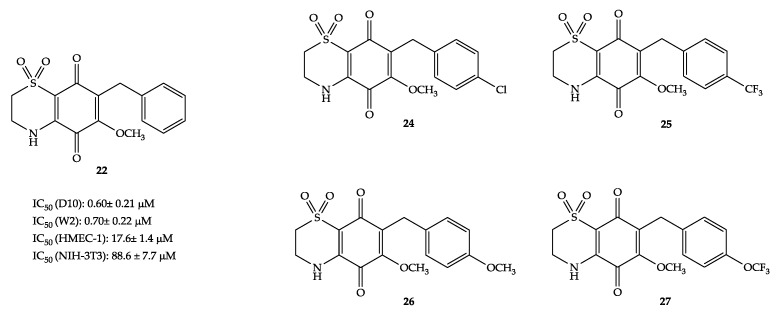
Structures of the *p*-substituted benzyl methoxy thiazinoquinones **24**–**27**.

Several antimalarial agents (e.g., artemisinin and its derivatives, chloroquine, mefloquine) are also effective against other parasites [16,17,18]. This prompted us to investigate the effects against *S. mansoni* of benzyl methoxy thiazinoquinone **22**, of the quinone **23**, and of the *ad hoc* synthesized *p*-substituted benzyl derivatives of **22**, compounds **24**–**27** (Figure 2). This study was performed in the frame of a wider screening aiming to explore the potential of the methoxy thiazinoquinone system to search for novel multi-stage antischistosomal agents [8,9]. In particular, the influence on the anti-parasitic activity against *P. falciparum* D10 and W2 strains as well as mammalian cells of various electron-withdrawing and -donating groups (-Cl, -CF_3_, -OCH_3_, and -OCF_3_) at the *para* position of the benzyl side chain was evaluated.

Thiazinoquinones **24**–**27**, as well as the quinone **23**, were first tested on schistosomula, the parasite larval stage; then, their effects on the viability of adult worm pairs and juvenile parasites, as well as on egg production and development, were evaluated. Interestingly, similar to what happens for the antiplasmodial effect, compounds **23** and all the regioisomeric A forms of methoxy thiazinoquinones were inactive [8]. All regioisomers B of *p*-substituted benzyl methoxy thiazinoquinones (compounds **24**–**27**) were active on both parasite forms and eggs, with compounds **26** and **27** being the most potent compounds in the series. In the present communication, a brief upgrade on the pharmacological effects of compounds **24**–**27** is reported. Herein, we describe the not yet reported antiplasmodial behavior of compounds **24**–**27** on D10 and W2 *P. falciparum* strains in vitro and their cytotoxicity against human microvascular endothelial cells (HMEC-1). These results, integrated with those previously reported on the schistosomicidal activity of these compounds, highlight compound **26** as a potential hit active against both *P. falciparum* and *S. mansoni*.

## 2. Results and Discussions

Thiazinoquinones **24**–**27** (Figure 2) were prepared from the commercially available 1,2,4-trimethoxybenzene as previously described [6,8]. In these compounds, the lead candidate **22** was modified by introducing various substituents (both electron-withdrawing and -donating groups) in the *para* position of the benzyl side chain. This chemical manipulation aimed to evaluate the possible effects on the antiparasitic activity and thus provide further evidence on the mode of action of thiazinoquinone derivatives. In our previously reported screening against *S. mansoni*, compounds **26** and **27**, both bearing an oxygen atom directly linked to the benzyl side chain, showed a better overall activity profile against the different forms of the parasite (larval, adult and juvenile forms, eggs) when compared to **22** [8]. The higher activity of these two derivatives has been supposed to be related to the presence of the H-bonds acceptor groups in the *para* position rather than the electronic effects on the aromatic ring of the substituents. Indeed, it has been supposed that these groups could possibly play a crucial role in the active thiazinoquinones, hypothesizing their membrane-transport proteins’ exploitation to enter the parasite [8]. Likewise, the schistosomicidal drug of choice praziquantel (PZQ) has been proposed to compete with adenosine for its transport into the parasite by binding to the nucleoside transporter, and SARs performed on PZQ indicated that its substitution with an aromatic moiety is detrimental, unless it is a phenyl ring substituted in *para* by a H-bond donor/acceptor group [8,19].

In order to evaluate the influence on the antimalarial activity of the substituents placed on the aromatic side chain of the lead candidate **22**, compounds **24**–**27** were tested against chloroquine (CQ)-sensitive (D10) and -resistant (W2) strains of *P. falciparum* by using the parasite lactate dehydrogenase (pLDH) assay [20]. All the *p*-substituted benzyl derivatives of **22** retained activity and were effective as antiplasmodial in the micromolar range. Moreover, compounds **24**–**27** were evaluated for their cytotoxic effects against HMEC-1 cells, too. The IC_50_ values on both *P. falciparum* strains and HMEC-1 cell line obtained for compounds **24**–**27**, as well as those of the lead compound **22**, are reported in Table 1. For each compound, the selectivity index (SI), namely the ratio between the IC_50_ values on the human cells and that on the parasite strains, was calculated; these data are reported in Table 1, alongside those calculated considering the IC_50_ values previously reported for compounds **22** and **24**–**27** on embryonic murine fibroblasts (NIH-3T3 cell line) [8]. It can be observed that, for the antiplasmodial activity, the IC_50_ values of compounds **24**–**27** are not significantly different from that of the lead candidate **22** and, actually, the activity of all thiazinoquinones was slightly decreased. The most remarkable lowering of the antiplasmodial activity was observed for compound **25**, which featured a substituent (-CF_3_) with a strong electron-withdrawing inductive effect at the *para* position of the aromatic ring and no resonance effect. On the other hand, the groups introduced at the *para* position of the benzyl ring of compounds **24**, **26**, and **27** (Figure 2), which showed lower IC_50_ values than **25** (Table 1), although characterized in some cases by opposite inductive effects, share the presence of a lone pair, which can be conjugated to the aromatic ring. Noteworthy, the potency of compound **27** was higher against the chloroquine-resistant W2 strain than against the D10 strain (see Table 1), with an IC_50_ in the sub-micromolar concentration range (IC_50_ = 0.81 µM).

Definitively, as for the antiplasmodial activity, modification of the lead compound **22** did not provide a better candidate. However, this study demonstrated that *p*-OCH_3_ and *p*-OCF_3_ benzyl derivatives of **22**, compounds **26** and **27**, which have been highlighted as new promising schistosomicidal multi-stage hits, both possess good antiplasmodial activity in the micromolar range, with compound **27** being more active among the two on the CQ-resistant strain W2.

As for the toxic effects exerted by compounds **26** and **27** on the cell growth of HMEC-1 (Table 1), compound **27** (cLogD = 1.37) [8] was the most cytotoxic thiazinoquinone in the tested series with an IC_50_ value of 3.59 µM. On the contrary, the thiazinoquinone **26** (cLogD = 0.33) [8] exhibited low cytotoxicity, comparable to that of the lead candidate **22** (cLogD = 0.41, Table 1). Furthermore, compound **26**, when tested on the NIH-3T3 cell line, was completely ineffective (IC_50_ > 100 µM) [8].

Since it is known that a cLogD value > 1 is needed for drugs’ passive diffusion in mammalian cells [21], then, the toxicity against HMEC-1 and NIH-3T3 cell lines showed by **22**, **26**, and **27** may be related to their cLogD values (Table S1), in line with previously reported SARs [6,8,9].

Evaluation of the calculated selectivity indexes (SIs) for the two different mammalian cell lines (see Table 1) of compounds **26** and **27** allowed a more proper assessment of their potential as antiparasitic agents. Even if their antiplasmodial and schistosomicidal potencies are comparable, compound **26** can be considered a better agent active against multiple parasites than **27** on the basis of its higher SI ratio on HMEC cells and its nontoxic effect on NIH-3T3 cells.

## 3. Materials and Methods

### 3.1. General Experimental Procedures

Commercial reagents and solvents: Sigma-Aldrich (Saint Louis, MO, USA) and Carlo Erba (Pomezia, Rome, Italy) whereas the anhydrous solvents were provided by Sigma-Aldrich or prepared by distillation according to standard procedures. Qualitative and semiquantitative TLC analyses: silica Gel 60 F254 (plates 5 × 20, 0.25 mm) and silica Gel 60 F254 plates (20 × 20.2 mm), respectively, Merck (Kenilworth, NJ, USA). Spots were revealed by a UV lamp, and then by spraying with 2 N sulfuric acid and heating at 120 °C. High-resolution electrospray ionization-mass spectrometry (ESI-MS (positive mode)) was performed on a Thermo LTQ Orbitrap XL mass spectrometer (Thermo-Fisher, San Josè, CA, USA) recording the spectra by infusion into the ESI source using MeOH as solvent.

NMR analysis was performed by a Bruker Avance Neo (Billerica, MA, USA) 700 MHz (700 and 175 MHz for ^1^H and ^13^C NMR, respectively). Chemical shifts were referenced to the residual solvent signal (CDCl_3_: δ_H_ = 7.26 and *δ*_C_ = 77.0). Homonuclear ^1^H connectivity was determined by correlation spectroscopy (COSY) experiments; one-bond heteronuclear ^1^H-^13^C connectivity by the heteronuclear single quantum coherence (HSQC) experiment; and two- and three-bond ^1^H-^13^C connectivity by gradient-HMBC experiments optimized for a ^2,3^*J* value of 8 Hz [22,23]. High-performance liquid chromatography (HPLC) separations were performed both on a Shimadzu LC-10AT (Shimadzu, Milan, Italy) apparatus and on a Knauer K-501 apparatus (LabService Analytica s.r.l., Anzola dell’Emilia, Italy). Each instrument was equipped with a Knauer K-2301 RI detector.

### 3.2. Synthesis of the Compounds **24**–**27**

The synthetic procedure needed to obtain the several thiazinoquinones **24**–**27** was already reported in our previous work. In particular, the protocol provided a good outcome for our interested compounds, confirming its interesting adaptability to different substrates [14].

*Compound **24***: orange powder; *t*_R_ = 14 min (single peak). ^1^H NMR in CDCl_3_ and HRESIMS spectra are reported in Supporting Information (SI). HRESIMS: *m/z* [M + Na]^+^ calcd. for C_16_H_14_ClNO_5_SNa^+^: 390.0173, found: 390.0171.

*Compound **25**:* orange powder; *t*_R_ = 21 min (single peak). ^1^H NMR in CDCl_3_ and HRESIMS spectra are reported in Supporting Information (SI). HRESIMS: *m/z* [M + Na]^+^ calcd. for C_17_H_14_F_3_NO_5_SNa^+^: 424.0437, found: 424.0438.

*Compound **26***: dark orange powder; *t*_R =_ 22 min (single peak). ^1^H NMR in CDCl_3_ and HRESIMS spectra are reported in Supporting Information (SI)). HRESIMS: *m/z* [M + H]^+^ calcd. for C_17_H_18_NO_6_S^+^: 364.0849, found: 364.0845.

*Compound **27***: orange powder; *t*_R_ = 14 min (single peak). ^1^H NMR in CDCl_3_ and HRESIMS spectra are reported in Supporting Information (SI). HRESIMS: *m/z* [M + H]^+^ calcd. for C_17_H_15_F_3_NO_6_S^+^: 418.0555, found: 418.0556.

### 3.3. P. falciparum Cultures and Drug Susceptibility Assay

All reagents were from Sigma Aldrich, Milan, Italy unless indicated otherwise. As reported in the literature [20], the chloroquine-sensitive (D10) and chloroquine-resistant (W2) strains of *P. falciparum* were maintained in vitro at 5% hematocrit medium (constituted of human type Apositive red blood cells) in RPMI 1640 (EuroClone, Celbio, Italy) with the addition of 1% AlbuMax (Invitrogen, Milan, Italy), 0.01% hypoxanthine, 20 µM HEPES, and 2 µM glutamine. The resulting cultures were incubated at 37 °C in a mixture of standard gas with the following composition: 1% O_2_, 5% CO_2_, and 94% N_2_. Drug susceptibility assay was required to solubilize the tested compounds in DMSO, and subsequently, diluted with the medium until the required concentration in order to have a final DMSO concentration less than 1% that was not toxic for the parasites. Drugs were placed in 96 well flat-bottom microplates and a series of dilutions performed. Asynchronous cultures (parasitemia of 1–1.5%; 1% final hematocrit) were aliquoted into the plates and incubated for 72 h at 37 °C. The cultures were washed with PBS before the chemosensitivity assay while the parasite growth was determined spectrophotometrically (OD650) by measuring the parasite lactate dehydrogenase (pLDH) activity. These measurements were made according to the modified version of Makler’s method both in control and drug-tested cultures [20]. Antiplasmodial activity is expressed as 50% inhibitory concentrations (IC_50_), which means the dose of compounds necessary to inhibit cell growth by 50%. Each IC_50_ value is the mean standard deviation of at least three separate experiments performed in duplicate.

### 3.4. Cytotoxicity Assay

The HMEC-1 cell line immortalized by SV 40 large T antigen43 was maintained in MCDB 131 medium (Invitrogen, Milan, Italy) supplemented with 10% heat-inactivated fetal calf serum (HyClone, Celbio, Milan, Italy), 10 ng/mL of epidermal growth factor (Chemicon), 1 mg/mL of hydrocortisone, 2 µM glutamine, 100 U/mL of penicillin, 100 mg/mL of streptomycin, and 20 µM of Hepes buffer (EuroClone).

## 4. Conclusions

In this communication, our aim was to investigate the effects against *P. falciparum* strains and HMEC-1 cell line of a series of methoxy *p*-benzyl-substituted thiazinoquinones (**24**–**27**) designed from compound **22**, earlier pointed out as an antimalarial lead candidate. For compounds **24**–**27**, each modification on the benzyl ring linked to the quinone moiety did not enhance the antiplasmodial activity, which was decreased with respect to compound **22** but, in any case, preserved. In the series, it is worthy to note that the more compelling compounds were **26** and **27**, which showed IC_50_ values against *P. falciparum* in the micromolar and sub-micromolar range. In summary, the results obtained expanded our SAR studies on substituted thiazinoquinones as antiparasitic agents, indicating that antiplasmodial activity is not affected by the inductive effect but rather by the (electron donating) resonance effect of the introduced substituent at the *para* position of the benzyl ring. The achieved data are an integral part of our ongoing investigation to identify new suitable antiparasitic agents whose therapeutic potential could be exploited in co-infection processes caused by more than one human parasite. Indeed, the current survey was based on the evaluation of antiparasitic usefulness as well as the selectivity on mammalian cells of the thiazinoquinones **24**–**27**, hence upgrading the knowledge about the active thiazinoquinone scaffold.

Considering the overall obtained antiplasmodial effects, the reported properties against all stages of the blood-dwelling *S. mansoni* [8], and the interference on the mammalian cells’ viability (HMEC-1 and NIH-3T3), we might define compound 26 as a new promising dual-acting antiparasitic agent since it showed itself as being superior among antiparasitic and antiproliferative effects. Finally, these studies corroborated the interesting potential of the bicyclic 1,1-dioxo-1,4-thiazine moiety as an active chemotype effective on more than one neglected parasite and, hence, able to vanquish the serious problem of the co-infection that is currently the cause of a high morbidity percentage in the most vulnerable countries worldwide.

## Figures and Tables

**Table 1:** IC_50_ in vitro values of compound **22** and **24**–**27** on chloroquine (CQ) ^a^ sensible (D10) ^b^ and resistant (W2) ^b^ strains of *P. falciparum*; cytotoxicity on HMEC-1 cell line ^c^ and relevant selectivity index (SI) ^d^; SI on NIH-3T3 cell line ^e^.

Compounds	D10 IC_50_ (µM)	W2 IC_50_ (µM)	HMEC-1 IC_50_ (µM) ^c^	SI ^d^		SI ^e^	
				D10	W2	D10	W2
**22**	0.60 ± 0.21	0.70 ± 0.22	17.6 ± 1.4	29.3	25.2	147.7	126.5
**24**	1.63 ± 0.35	1.90 ± 1.05	8.51 ± 0.76	5.2	4.5	48.5	41.6
**25**	5.06 ± 1.68	5.02 ± 1.81	20.4 ± 1.60	4.0	4.1	11.0	11.1
**26**	1.21 ± 0.20	1.22 ± 0.24	17.9 ± 1.10	14.8	14.7	– ^f^	– ^f^
**27**	1.12 ± 0.40	0.81 ± 0.19	3.59 ± 0.72	3.2	4.4	32.6	45.0

^a^ CQ as positive control: D10 IC_50_ (μM) = 0.04 ± 0.01; W2 IC_50_ (μM) = 0.54 ± 0.28; not cytotoxic. ^b^ Data are the mean ± SD of three different experiments in duplicate. ^c^ Camptothecin as positive control: IC_50_ (μM) = 0.018 ± 0.008. ^d^ SI = IC_50_ HMEC-1/IC_50_ *P. falciparum* strain. ^e^ SI = IC_50_ NIH-3T3/IC_50_ *P. falciparum* strain; cytotoxicity values on NIH-3T3 (µM) of compounds **22** and **24**–**27** are reported in a previous work [8]. ^f^ SI was not calculated since **26** is not active on NIH-3T3.

## Supplementary Materials

The following are available online at https://www.mdpi.com/1420-3049/25/7/1530/s1, Figure S1: HRESIMS spectrum of compound **24**, Figure S2: ^1^H NMR spectrum of compound **24** in CDCl_3_, Figure S3: HRESIMS spectrum of compound **25**, Figure S4: ^1^H NMR spectrum of compound **25** in CDCl_3_, Figure S5: HRESIMS spectrum of compound **26**, Figure S6: ^1^H NMR spectrum of compound **26** in CDCl_3_, Figure S7: HRESIMS spectrum of compound **27**, Figure S8: ^1^H NMR spectrum of compound **27** in CDCl_3_, Table S1: cLogD values of compounds **22** and **24–27**.
