# Supplementary material for: Antiplasmodial Activity of p-Substituted Benzyl Thiazinoquinone Derivatives and Their Potential against Parasitic Infections
Source: Molecules. 2020 Mar 27;25(7):1530. doi: 10.3390/molecules25071530 (PMC7180939; doi:10.3390/molecules25071530)
Supplement: Supplementary file 1 [file molecules-25-01530-s001.pdf]

Antiplasmodial activity of *p*-substituted benzyl thiazinoquinone derivatives and their potential against multiparasitic infections

Marcello Casertano[1,2], Marialuisa Menna[1,2], Caterina Fattorusso[1,2], Nicoletta Basilico[3], Silvia Parapini[4], Marco Persico[1,2], and Concetta Imperatore[1, 2,*]

[1] Department of Pharmacy, University of Naples "Federico II", Via D. Montesano 49, 80131 Napoli, Italy; marcello.casertano@unina.it (M.C.); mlmenna@unina.it (M.M.); caterina.fattorusso@unina.it (C.F.); marco.persico@unina.it (M.P.);

[2] Italian Malaria Network, Centro Interuniversitario di Ricerche Sulla Malaria (CIRM), Department of Pharmacy, University of Naples "Federico II", Via D. Montesano 49, 80131 Napoli, Italy;

[3] Dipartimento di Scienze Biomediche, Chirurgiche e Odontoiatriche, Università di Milano, Via Pascal 36, 20133 Milan, Italy; nicoletta.basilico@unimi.it (N.B.);

[4] Dipartimento di Scienze Biomediche per la Salute, Università di Milano, Via Pascal 36, 20133 Milan, Italy; silvia.parapini@unimi.it (S.P.);

* Correspondence: cimperat@unina.it (C.I.); Tel.: +39081678530.

TABLE OF CONTENTS

Figure S1. HRESIMS spectrum of compound **24** 3

Figure S2. ^{1}H NMR spectrum of compound **24** in $CDCl_3$ 3

Figure S3. HRESIMS spectrum of compound **25** 4

Figure S4. ^{1}H NMR spectrum of compound **25** in $CDCl_3$ 4

Figure S5. HRESIMS spectrum of compound **26** 5

Figure S6. ^{1}H NMR spectrum of compound **26** in $CDCl_3$ 5

Figure S7. HRESIMS spectrum of compound **27** 6

Figure S8. ^{1}H NMR spectrum of compound **27** in $CDCl_3$ 6

Table S1. cLogD values of compounds **22** and **24-27** 7

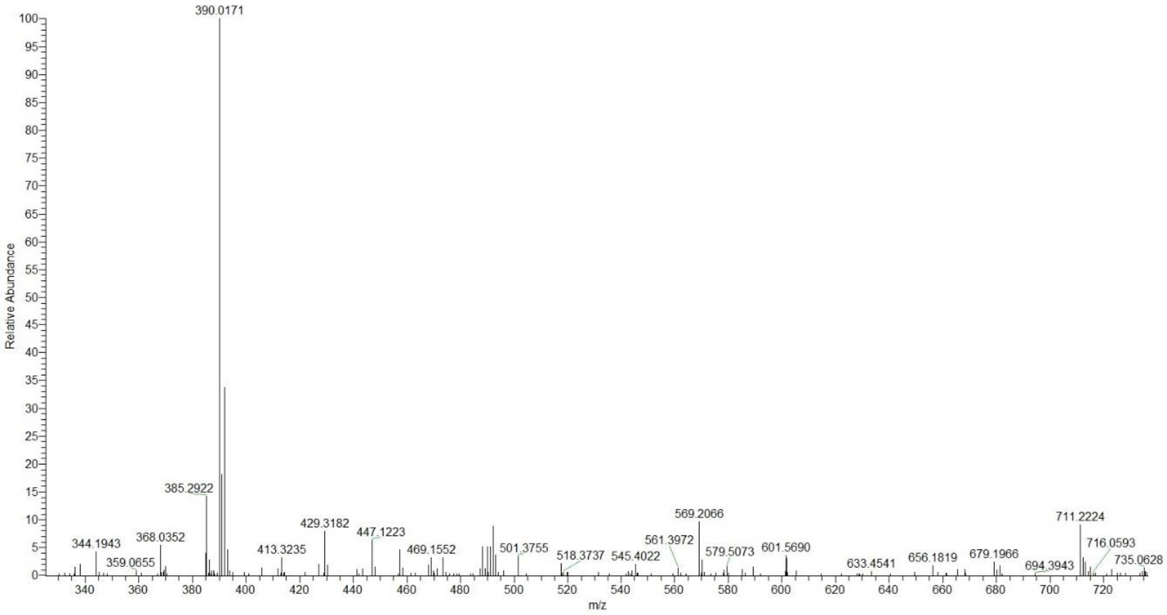

Figure S1. HRESIMS spectrum of compound **24**

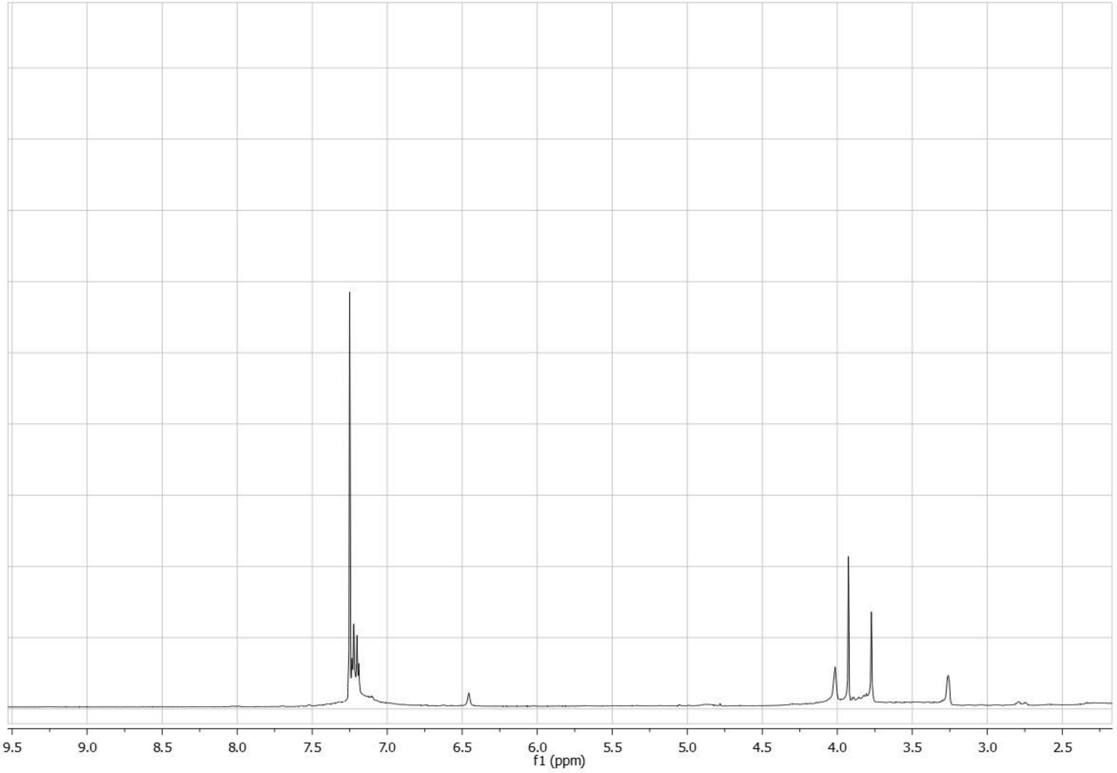

Figure S2. ^{1}H NMR spectrum of compound **24** in $CDCl_3$

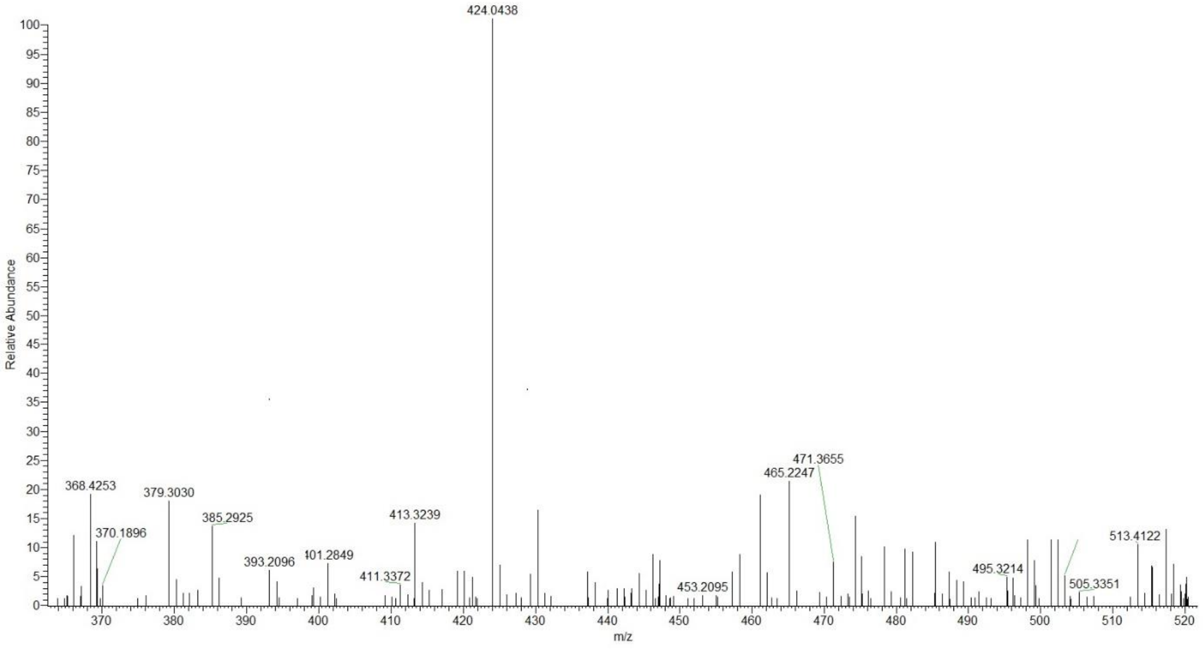

Figure S3. HRESIMS spectrum of compound **25**

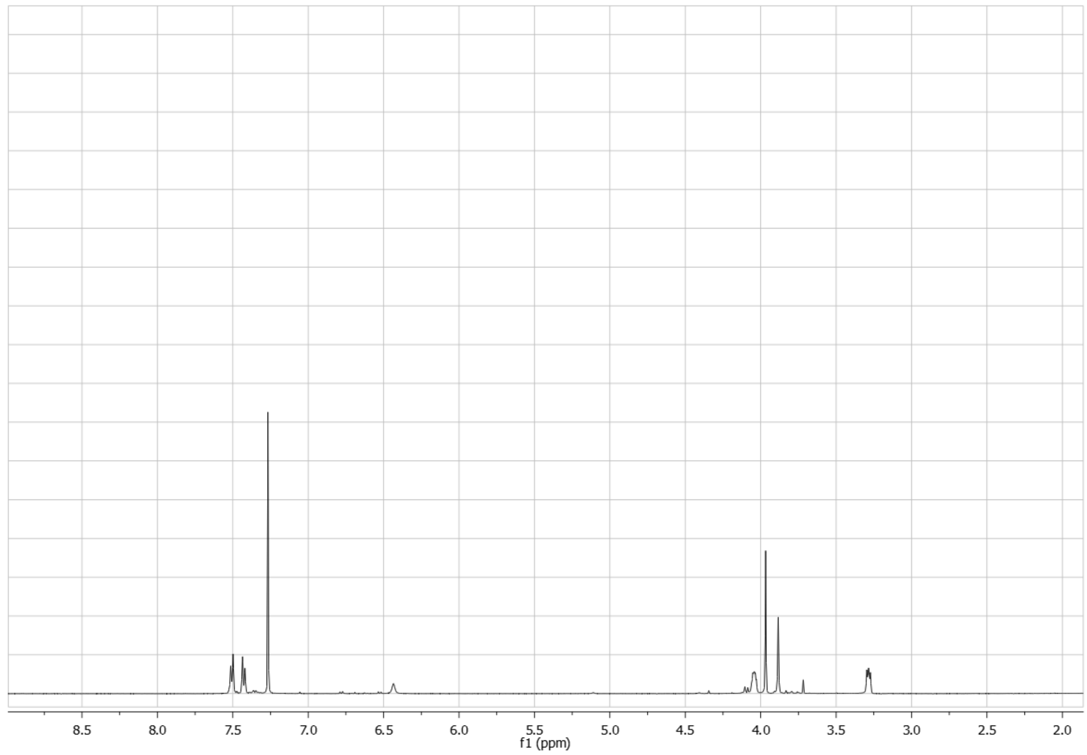

Figure S4. ^{1}H-NMR spectrum of compound **25** in $CDCl_3$

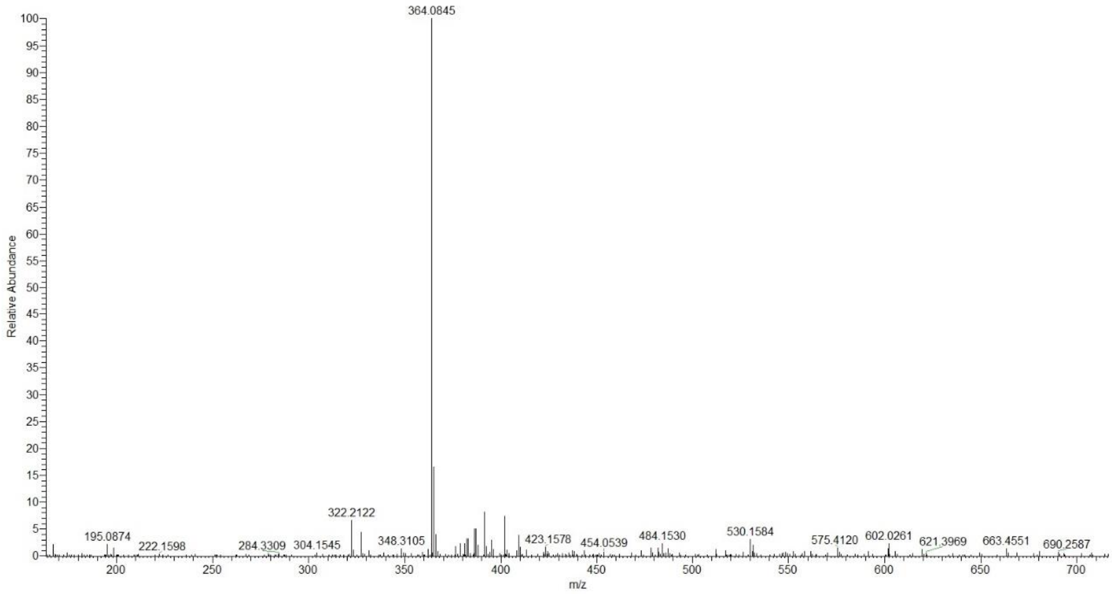

Figure S5. HRESIMS spectrum of compound **26**

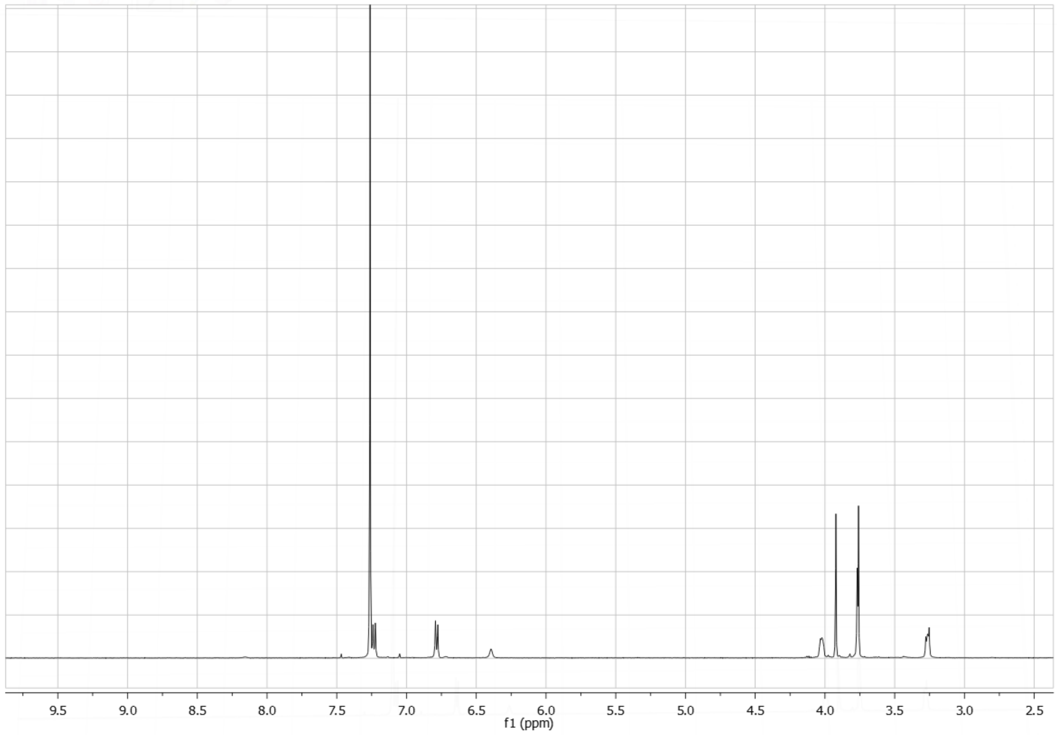

Figure S6. ^{1}H-NMR spectrum of compound **26** in $CDCl_3$

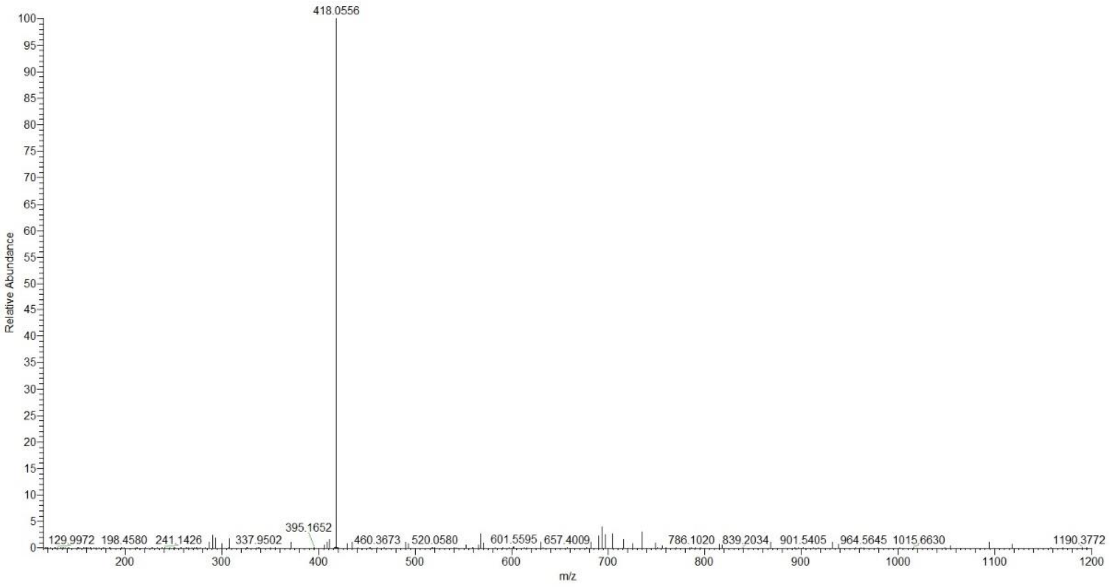

Figure S7. HRESIMS spectrum of compound **27**

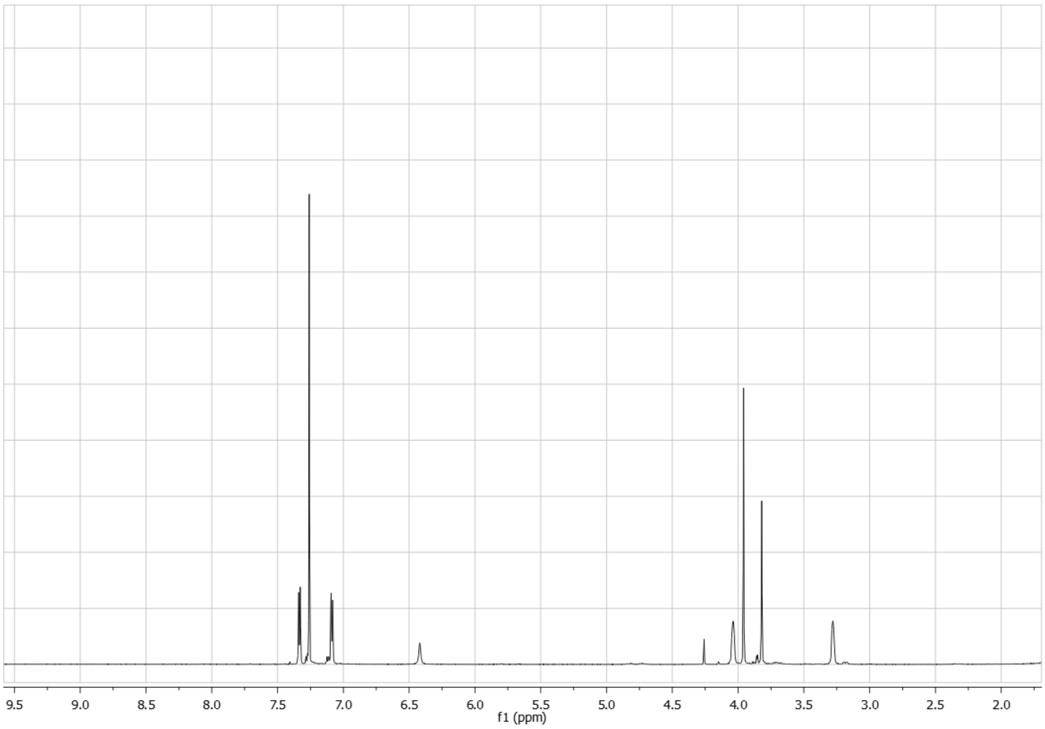

Figure S8. ^{1}H-NMR spectrum of compound **27** in $CDCl_3$

Table S1. cLogD values of compounds **22** and **24-27**

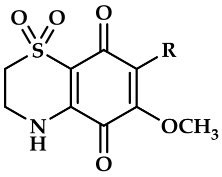

Compound	R	cLogD[a]
**22**	$-CH_2C_6H_5$	0.41
**24**	$-CH_2C_6H_4$-*p*Cl	1.01
**25**	$-CH_2C_6H_4$-*p*CF_3	0.99
**26**	$-CH_2C_6H_4$-*pO*CH_3	0.33
**27**	$-CH_2C_6H_4$-*pO*CF_3	1.37

[a]cLogD calculated considering pH values: 7.4 and 7.2.
